# EEGformer: A transformer–based brain activity classification method using EEG signal

**DOI:** 10.3389/fnins.2023.1148855

**Published:** 2023-03-24

**Authors:** Zhijiang Wan, Manyu Li, Shichang Liu, Jiajin Huang, Hai Tan, Wenfeng Duan

**Affiliations:** ^1^The First Affiliated Hospital of Nanchang University, Nanchang University, Nanchang, Jiangxi, China; ^2^School of Information Engineering, Nanchang University, Nanchang, Jiangxi, China; ^3^Industrial Institute of Artificial Intelligence, Nanchang University, Nanchang, Jiangxi, China; ^4^School of Computer Science, Shaanxi Normal University, Xi’an, Shaanxi, China; ^5^Faculty of Information Technology, Beijing University of Technology, Beijing, China; ^6^School of Computer Science, Nanjing Audit University, Nanjing, Jiangsu, China

**Keywords:** brain activity classification, SSVEPs, EEGformer, EEG characteristics, deep learning

## Abstract

**Background:**

The effective analysis methods for steady-state visual evoked potential (SSVEP) signals are critical in supporting an early diagnosis of glaucoma. Most efforts focused on adopting existing techniques to the SSVEPs-based brain–computer interface (BCI) task rather than proposing new ones specifically suited to the domain.

**Method:**

Given that electroencephalogram (EEG) signals possess temporal, regional, and synchronous characteristics of brain activity, we proposed a transformer–based EEG analysis model known as EEGformer to capture the EEG characteristics in a unified manner. We adopted a one-dimensional convolution neural network (1DCNN) to automatically extract EEG-channel-wise features. The output was fed into the EEGformer, which is sequentially constructed using three components: regional, synchronous, and temporal transformers. In addition to using a large benchmark database (BETA) toward SSVEP-BCI application to validate model performance, we compared the EEGformer to current state-of-the-art deep learning models using two EEG datasets, which are obtained from our previous study: SJTU emotion EEG dataset (SEED) and a depressive EEG database (DepEEG).

**Results:**

The experimental results show that the EEGformer achieves the best classification performance across the three EEG datasets, indicating that the rationality of our model architecture and learning EEG characteristics in a unified manner can improve model classification performance.

**Conclusion:**

EEGformer generalizes well to different EEG datasets, demonstrating our approach can be potentially suitable for providing accurate brain activity classification and being used in different application scenarios, such as SSVEP-based early glaucoma diagnosis, emotion recognition and depression discrimination.

## 1. Introduction

Glaucoma is known as a “silent thief of sight,” meaning that patients do not notice the health condition of their visual function until vision loss and even blindness occur (Abdull et al., 2016). According to the world health organization, the number of people with glaucoma worldwide in 2020 is 76 million, and the patient number would be increased to 95.4 million in 2030. As the population ages, the number with this condition will also increase substantially (Guedes, 2021). Glaucoma causes irreversible optic nerve vision damage. It is crucial to provide accurate early screening to diagnose patients in their early stages so that they can receive appropriate early treatment. Steady-state visual evoked potentials (SSVEPs), which refer to a stimulus-locked oscillatory response to periodic visual stimulation commonly exerted in the visual pathway of humans, can be used to evaluate the functional abnormality of the visual pathway that is essential for the complete transmission of visual information (Zhou et al., 2020). SSVEPs are always measured using electroencephalogram (EEG) measurement and have been widely used in the study of brain–computer interface (BCI). Because peripheral vision loss is a key diagnostic sign of glaucoma, patients cannot be evoked by certain repetitive stimuli with a constant frequency from vision loss regions (Khok et al., 2020). Therefore, stimuli with the corresponding frequency are not detected by the primary visual cortex. Thus, the SSVEPs-based BCI applications can be used in the early diagnosis of visual function detection for patients with glaucoma.

The effective analysis method for SSVEPs is critical in the accurate early diagnosis of glaucoma. SSVEPs are EEG activity with a spatial-spectral-temporal (SST) pattern. It is easy to understand that SSVEP signals, such as the EEG signal measured over time, could be analyzed using time series analysis methods. Brain functional connectivity (BFC) can be used to capture spatial patterns from multiple brain regions by analyzing the correlations between brain activities detected from different regions. The spectral pattern extraction method is the most popular method for analyzing the frequency characteristics of EEG signals. For instance, power spectra density–based analysis (PSDA) is a commonly used frequency detection method that can classify various harmonic frequencies from EEG signals (Zhang et al., 2020). In addition, canonical correlation analysis (CCA) (Zhuang et al., 2020) and other similar algorithms, such as multivariate synchronization index (MSI) (Qin et al., 2021) and correlated component analysis (COCA) (Zhang et al., 2019), are effective frequency detection algorithms based on the multivariate statistical analysis method. Although SST pattern extraction algorithms have demonstrated satisfactory results, most patterns or features extracted from raw EEG data require a manually predefined algorithm based on expert knowledge. The procedure of learning handcrafted features for SSVEP signals is not flexible and might limit the performance of these systems in brain activity analysis tasks.

In recent years, deep learning (DL) methods have achieved excellent performance in processing EEG-based brain activity analysis tasks (Li Z. et al., 2022; Schielke and Krekelberg, 2022). Currently, the mainstream technologies of using DL to process SSVEP signal could be divided into two aspects: convolutional neural network (CNN) based methods and transformer-based methods. For the CNN-based methods, Li et al. (2020) propose a CNN-based nonlinear model, i.e. convolutional correlation analysis (Conv-CA), to transform multiple channel EEGs into a single EEG signal and use a correlation layer to calculate correlation coefficients between the transformed single EEG signal and reference signals. Guney et al. (2021) propose a deep neural network architecture for identifying the target frequency of harmonics. Waytowich et al. (2018) design a compact convolutional neural network (Compact-CNN) for high-accuracy decoding of SSVEPs signal. For the transformer-based methods, Du et al. (2022) propose a transformer-based approach for the EEG person identification task that extracts features in the temporal and spatial domains using a self-attention mechanism. Chen et al. (2022) propose SSVEPformer, which is the first application of the transformer to the classification of SSVEP. Li X. et al. (2022) propose a temporal-frequency fusion transformer (TFF-Former) for zero-training decoding across two BCI tasks. The aforementioned studies demonstrate the competitive model performance of DL methods in performing SSVEPs-based BCI tasks. However, most existing DL efforts focused on applying existing techniques to the SSVEPs-based BCI task rather than proposing new ones specifically suited to the domain. Standard well-known network architectures are designed for data collected in natural scenes and do not consider the peculiarities of the SSVEP signals. Therefore, further research is required to understand how these architectures can be optimized for EEG-based brain activity data.

The main question is what is the specificity of the SSVEP signal analysis domain and how to use machine learning methods (particularly DL methods) to deal with the signal characteristics. Because the SSVEP signal is EEG-based brain activity, we can answer the question by analyzing the EEG characteristics in the brain activity analysis domain. Specifically, EEG characteristics are reflected in three aspects: temporal, regional, and synchronous characteristics. The temporal characteristics (e.g., mean duration, coverage, and frequency of occurrence) are easily traceable in standard EEG data and provide numerous sampling points in a short time (Zhang et al., 2021), thereby providing an efficient way to investigate trial-by-trial fluctuations of functional spontaneous activity. The regional characteristics refer to different brain regions that are linked to distinct EEG bands (Nentwich et al., 2020). The synchronous characteristics refer to the synchronous brain activity pattern over a functional network including several brain regions with similar spatial orientations (Raut et al., 2021). Traditionally, brain response to a flickering visual stimulation has been considered steady-state, in which the elicited effect is believed to be unchanging in time. In fact, the SSVEPs belongs to a signal with non-stationary nature, which indicates dynamical patterns and complex synchronization between EEG channels can be used to further understand brain mechanisms in cognitive and clinical neuroscience. For instance, Ibáñez-Soria et al. explored the dynamical character of the SSVEP response by proposing a novel non-stationary methodology for SSVEP detection, and found dynamical detection methodologies significantly improves classification in some stimulation frequencies (Ibáñez-Soria et al., 2019). Tsoneva et al. (2021) studied the mechanisms behind SSVEPs generation and propagation in time and space. They concluded that the SSVEP spatial properties appear sensitive to input frequency with higher stimulation frequencies showing a faster propagation speed. Thus, we hypothesize that a machine learning method that can capture the EEG characteristics in a unified manner can suit the SSVEPs-based BCI domain and improve the model performance in EEG-based brain activity analysis tasks.

In this study, we propose a transformer–based EEG analysis model known as the EEGformer (Vaswani et al., 2017) to capture the EEG characteristics in the SSVEPs-based BCI task. The EEGformer is an end-to-end DL model, processing SSVEP signals from the EEG to the prediction of the target frequency. The component modules of the EEG former are depicted as follows:

(1) Depth-wise convolution-based one-dimensional convolution neural network (1DCNN). The depth-wise convolution-based 1DCNN is first used to process the raw EEG input. Assuming the raw data is collected from *C* EEG channels, there are *M* depth-wise convolutional filters for generating *M* feature maps. Each convolutional filter is responsible for shifting across the raw data in an EEG-channel-wise manner and extracting convolutional features from the raw data of each EEG channel to form a feature map. Unlike other techniques that manually extract temporal or spectrum features based on the time course of the EEG signal, we use the depth-wise convolutional filter to extract the EEG features in a completely data–driven manner. Because the feature map is generated by the same depth-wise convolutional filter, each row of the feature map shares the same convolutional property. Follow-up convolutional layers are allocated with several depth-wise convolutional filters to enrich the convolutional features and deepen the 1DCNN network. A three-dimensional (3D) feature matrix is used to represent the output of the 1DCNN network. The *x*, *y*, and *z* dimensions of the 3D feature matrix represent temporal, spatial, and convolutional features, respectively.
(2) EEGformer encoder. This component module consists of three sub-modules: temporal, synchronous, and regional transformers, which are used in learning the temporal, synchronous, and regional characteristics, respectively. The core strategy of learning EEG characteristics by our model mainly include two steps: input tokens that serve as the basic elements of learning the temporal, synchronous, and regional characteristics are sliced from the 3D feature matrix along the temporal, convolutional, and spatial dimension, respectively. And then, self-attention mechanism is employed to measure the relationships between pairs of input tokens and give tokens more contextual information, yielding more powerful features for representing the EEG characteristics. The three components could be performed in a sequential computing order, allowing the encoder to learn the EEG characteristics in a unified manner.
(3) EEGformer decoder. This module contains three convolutional layers and one fully connected (FC) layer. The output of the last FC layer is fed to a softmax function which produces a distribution over several category labels. The categorical cross entropy combined with regularization was used as the loss function for training the entire EEGformer pipeline. The EEGformer decoder is used to deal with specific tasks, such as target frequency identification, emotion recognition, and depression discrimination. In addition to using a large benchmark database (BETA) (Liu et al., 2020) to validate the performance of the SSVEP-BCI application, we validate the model performance on two additional EEG datasets, one for emotion analysis using EEG signals [SJTU emotion EEG dataset (SEED)] (Duan et al., 2013; Zheng and Lu, 2015) and the other for a depressive EEG database (DepEEG) (Wan et al., 2020) obtained from our previous study, to support our hypothesis that highlights the significance of learning EEG characteristics in a unified manner for EEG-related data analysis tasks.

The main contributions of this study are as follows: (1) current mainstream DL models have superior ability in processing data collected in natural scenes and might not adept at dealing with SSVEP signals. To achieve a DL model that can be applied to the specificity of the SSVEP signal analysis domain and obtain better model performance in SSVEPs-based frequency recognition task, we propose a transformer–based EEG analysis model known as the EEGformer to capture the EEG characteristics in a unified manner. (2) To obtain a flexible method for addressing the SSVEPs-based frequency recognition and avoid the model performance limited by manual feature extraction, we adopt 1DCNN to automatically extract EEG-channel-wise features and fed them into the EEGformer. This operation transforms our method into a complete data–driven manner for mapping raw EEG signals into task decisions. (3) To ensure the effectiveness and generalization ability of the proposed model, we validate the performance of the EEGformer on three datasets for three different EEG-based data analysis tasks: target frequency identification, emotion recognition, and depression discrimination.

## 2. Materials and methods

### 2.1. Dataset preparation

Table 1 shows some detailed information about the three datasets (BETA, SEED, and DepEEG) that we used as benchmarks to validate the effectiveness of this study. The participants’ column in the table describes how many subjects joined in the corresponding data collection. The experiment per participant (EPP) column shows how many experiments were performed by each participant. The trails per experiment (TPE) column shows how many trails are executed in one experiment. The channel number (CHN) column shows the CHN of the EEG dataset. The sampling rate (SR) column shows the down-sampling rate of the EEG signal. The time length per trail (TLPT) column shows the time length of a single trail in seconds. The labels column shows the categorical emotion labels for the classification task and emotional intensity for the regression task. Specifically, for the target frequency identification task, we classified 40 categories of harmonic frequencies and the frequency range is 8–15.8 HZ with 0.2 HZ intervals. For the emotion recognition task, we used arousal, valence, and dominance rating scores as the dataset labels. For the depression discrimination task, we classified EEG samples from depressive or normal control.

**TABLE 1:** Detail information on the three datasets.

Dataset	Participants	EPP	TPE	CHN	SR (HZ)	Labels	TLPT
BETA	70 healthy subjects	4	40	64	250	40 harmonics, e.g., f_j_ ∈ {8,8.2,…,15.8}	2/3 s
SEED	15 healthy subjects	3	15	62	200	Positive, neutral, negative	305 s
DepEEG	12 healthy subjects and 23 depressives	1	1	6	500	Depressive, normal control	≥480 s

### 2.2. Pipeline of EEGformer–based brain activity analysis

**FIGURE 1:**
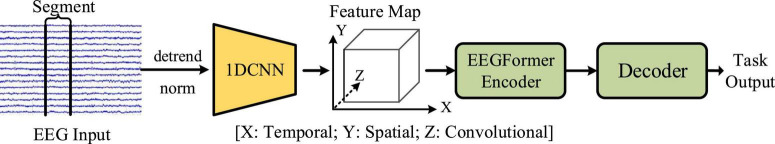
Pipeline of EEGformer for different tasks of brain activity analysis.

Figure 1 shows the pipeline of EEGformer–based brain activity analysis. The core modules of the pipeline include 1DCNN, EEGformer encoder, and decoder. The input of the 1DCNN is an EEG segment represented using a two-dimensional (2D) matrix of size *S* × *L*, where *S* represents the number of EEG channels, and *L* represents the segment length. The EEG segment is de-trend and normalized before being fed into the 1DCNN module, and the normalized EEG segment is represented by x ∈ *R^S × L^*. The 1DCNN adopts multiple depth-wise convolutions to extract EEG-channel-wise features and generate 3D feature maps. It shifts across the data along the EEG channel dimension for each depth-wise convolution and generates a 2D feature matrix of size *S* × *L*_*f*_, where *L*_*f*_ is the length of the extracted feature vector. The output of the 1DCNN module is a 3D feature matrix of size *S* × *C* × *L*_*e*_, where *C* is the number of depth-wise convolutional kernels used in the last layer of the 1DCNN module, *L*_*e*_ is the features length outputted by the last layer of the 1DCNN module. More specifically, the 1DCNN is comprised of three depth-wise convolutional layers. Hence, we have the processing x → z_1_ → z_2_ → z_3_, where z_1_, z_2_, and z_3_ denote the outputs of the three layers. The size of the depth-wise convolutional filters used in the three layers is 1 × 10, valid padding mode is applied in the three layers and the stride of the filters is set to 1. The number of the depth-wise convolutional filter used in the three layers is set to 120, ensuring sufficient frequency features for learning the regional and synchronous characteristics. We used a 3D coordinate system to depict the axis meaning of the 3D feature matrix. The *X*, *Y*, and *Z* axes represent the temporal, spatial, and convolutional feature information contained in the 3D feature matrix, respectively. The output of the 1DCNN module is fed into the EEGformer encoder for encoding the EEG characteristics (regional, temporal, and synchronous characteristics) in a unified manner. The decoder is responsible for decoding the EEG characteristics and inferencing the results according to the specific task.

### 2.3. EEGformer encoder

**FIGURE 2:**
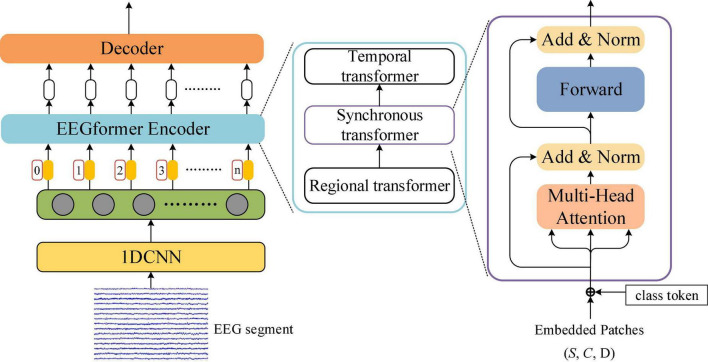
Illustration of the EEGformer architecture.

The EEGformer encoder is used to provide a uniform feature refinement for the regional, temporal, and synchronous characteristics contained in the output of the 1DCNN module. Figure 2 illustrates the EEGformer architecture and shows that the EEGformer encoder uses a serial structure to sequentially refine the EEG characteristics. The temporal, regional, and synchronous characteristics are refined using temporal, regional, and synchronous transformers, respectively. The outputs of the 1DCNN are defined as *z*_3_ ∈ *R*^S×*C*×*L*_*e*_^ and are represented using black circles in the green rectangle box.

The specific computing procedures of each transformer module are depicted as follows:

#### 2.3.1. Regional transformer module

The input of the regional transformer module is represented by *z*_3_ ∈ *R*^C×*L*_*e*_×*S*^. The 3D matrix *z_3_* is first segmented into *S* 2D submatrices along the spatial dimension. Each submatrix is represented by ${\text{X}}_{i}^{r\,e\,g}\in R^{C\times L_{e}}$ (*i* = 1,2,3,…,*S*). The input of the regional transformer module is represented by *S* black circles in the green rectangle box and each circle represents a submatrix. The vector $X_{\left(i,c\right)}^{r\,e\,g}\in R^{L_{e}}$ is sequentially taken out from the ${\text{X}}_{i}^{r\,e\,g}$ along the convolutional feature dimension and fed into the linear mapping module. According to the terminology used in the vision of transformer (ViT) studies, we defined the vector $X_{\left(i,c\right)}^{r\,e\,g}$ as a patch of the regional transformer module. Each $X_{\left(i,c\right)}^{r\,e\,g}$ is represented by a tiny yellow block in the Figure 2. The $X_{\left(i,c\right)}^{r\,e\,g}$ is linearly mapped into a latent vector $z_{\left(i,c\right)}^{\left(r\,e\,g,0\right)}\in R^{D}$ using a learnable matrix M ∈ *R*^D×*L*_*e*_^:


(1) $$z_{\left(i,c\right)}^{\left(r\,e\,g,0\right)}=\text{M}\,X_{\left(i,c\right)}^{r\,e\,g}+e_{\left(i,c\right)}^{p\,o\,s},$$

where $e_{\left(i,c\right)}^{p\,o\,s}\in R^{D}$ denotes a positional embedding added to encode the position for each convolutional feature changing over time. The regional transformer module also consists of *K* ≥ 1 encoding blocks, each block contains two layers: a multi-head self-attention layer and a position-wise fully connected feed-forward network. The resulting $z_{\left(i,c\right)}^{\left(r\,e\,g,0\right)}$ is defined as a token representing the inputs of each block, and the $z_{\left(0,0\right)}^{\left(r\,e\,g,0\right)}$ indicates the classification token. The *l*-th block produces an encoded representation $z_{\left(i,c\right)}^{\left(r\,e\,g,l\right)}$ for each token in the input sequence by incorporating the attention scores. Specifically, at each block *l*, three core vectors, including $q_{\left(i,c\right)}^{\left(l,a\right)}$, $k_{\left(i,c\right)}^{\left(l,a\right)}$, and $v_{\left(i,c\right)}^{\left(l,a\right)}$ are computed from the representation $z_{\left(i,c\right)}^{\left(r\,e\,g,l-1\right)}$ encoded by the preceding layer:


(2) $$q_{\left(i,c\right)}^{\left(l,a\right)}=W_{Q}^{\left(l,a\right)}\,L\,N\,\left(z_{\left(i,c\right)}^{\left(r\,e\,g,l-1\right)}\right)\in R^{D_{h}},$$

(3) $$k_{\left(i,c\right)}^{\left(l,a\right)}=W_{K}^{\left(l,a\right)}\,L\,N\,\left(z_{\left(i,c\right)}^{\left(r\,e\,g,l-1\right)}\right)\in R^{D_{h}},$$

(4) $$v_{\left(i,c\right)}^{\left(l,a\right)}=W_{V}^{\left(l,a\right)}\,L\,N\,\left(z_{\left(i,c\right)}^{\left(r\,e\,g,l-1\right)}\right)\in R^{D_{h}},$$

where $W_{Q}^{\left(l,a\right)}$, $W_{K}^{\left(l,a\right)}$, and $W_{V}^{\left(l,a\right)}$ are the matrixes of query, key, and value in the regional transformer module, respectively. LN() denotes the LayerNorm operation, and a ∈ {1, 2, 3, …, A} is an index over the multi-head self-attention units. A is the number of units in a block. *D_h_* is the quotient computed by D/A and denotes the dimension number of three vectors. The regional self-attention (RSA) scores for $z_{\left(i,c\right)}^{\left(r\,e\,g,l-1\right)}$ in the *a*-th multi-head self-attention unit is given as follows:


(5) α(i,c)(l,a)r⁢e⁢g=σ⁢(q(i,c)(l,a)Dh⋅[k(0,0)(l,a)⁢{k(i,c)(l,a)}c=1,…,C])∈RC,

where σ denotes the softmax activation function, and the symbol **⋅** denotes the dot product for computing the similarity between the query and key vectors. $k_{\left(i,c\right)}^{\left(l,a\right)}$ and $q_{\left(i,c\right)}^{\left(l,a\right)}$ represent the corresponding key and query vectors, respectively. The equation shows that the RSA scores are merely computed over convolutional features of single brain region. That is, the RSA can calculate the contribution of a changing mono-electrode convolutional feature to the final model decision at a specific EEG channel. An intermediate vector $s_{\left(i,c\right)}^{\left(l,a\right)}$ for encoding $z_{\left(i,c\right)}^{\left(r\,e\,g,l-1\right)}$ is given as follows:


(6) $$s_{\left(i,c\right)}^{\left(l,a\right)}=\alpha_{\left(i,0\right)}^{\left(l,a\right)}\,v_{\left(i,0\right)}^{\left(l,a\right)}+\sum_{j=1}^{C}\alpha_{\left(i,j\right)}^{\left(l,a\right)}\,v_{\left(i,j\right)}^{\left(l,a\right)}\in R^{D_{h}}.$$

The encoded feature $z_{\left(i\right)}^{\left(r\,e\,g,l\right)}\in R^{C\times D}$ by the *l*-th block is computed by first concatenating the intermediate vectors from all heads, and the vector concatenation is projected by matrix *W*_*O*_ ∈ *R*^D×*L*^, where *L* is equal to A=*D*_*h*_. z(i)(reg,l)′ is the residual connection result of the projection of the intermediate vectors and the $z_{\left(i\right)}^{\left(r\,e\,g,l-1\right)}$ encoded by the preceding block. Finally, the z(i)(reg,l)′ normalized by LN() is passed through a multilayer perceptron (MLP) using the residual connection. The output of the regional transformer is represented by *z*_4_ ∈ *R*^S×*C*×D^.

#### 2.3.2. Synchronous transformer module

The input of the synchronous transformer module is represented by *z*_4_ ∈ *R*^S×*L*_*e*_×*C*^. The 3D matrix *z_4_* is first segmented into *C* 2D submatrices along the convolutional feature dimension. Each submatrix is represented by ${\text{X}}_{i}^{s\,y\,n}\in R^{S\times D}$ (*i* = 1,2,3,…,*C*). The vector $X_{\left(i,s\right)}^{s\,y\,n}\in R^{D}$ is sequentially taken out from the ${\text{X}}_{i}^{s\,y\,n}$ along the spatial dimension and fed into the linear mapping module. The $X_{\left(i,s\right)}^{s\,y\,n}$ is defined as a patch and is linearly mapped into a latent vector $z_{\left(i,s\right)}^{\left(s\,y\,n,0\right)}\in R^{D}$ using a learnable matrix M ∈ *R*^D×*D*^:


(7) $$z_{\left(i,s\right)}^{\left(s\,y\,n,0\right)}=\text{M}\,X_{\left(i,s\right)}^{s\,y\,n}+e_{\left(i,s\right)}^{p\,o\,s},$$

where $e_{\left(i,s\right)}^{p\,o\,s}\in R^{D}$ denotes a positional embedding added to encode the spatial position for each EEG channel changing over time. The synchronous transformer also consists of *K* ≥ 1 encoding blocks, and each block contains two layers: a multi-head self-attention layer and a position-wise fully connected feed-forward network. The resulting $z_{\left(i,s\right)}^{\left(s\,y\,n,0\right)}$ is defined as a token representing the inputs of each block, and the $z_{\left(0,0\right)}^{\left(s\,y\,n,0\right)}$ indicates the classification token. The *l*-th block produces an encoded representation $z_{\left(i,s\right)}^{\left(s\,y\,n,l\right)}$ for each token in the input sequence by incorporating the attention scores. Specifically, at each block *l*, three core vectors, including $q_{\left(i,s\right)}^{\left(l,a\right)}$, $k_{\left(i,s\right)}^{\left(l,a\right)}$, and $v_{\left(i,s\right)}^{\left(l,a\right)}$ are computed from the representation $z_{\left(i,s\right)}^{\left(s\,y\,n,l-1\right)}$ encoded by the preceding layer:


(8) q(i,s)(l,a)=WQ(l,a)′⁢L⁢N⁢(z(i,s)(s⁢y⁢n,l-1))∈RDh,

(9) k(i,s)(l,a)=WK(l,a)′⁢L⁢N⁢(z(i,s)(s⁢y⁢n,l-1))∈RDh,

(10) v(i,s)(l,a)=WV(l,a)′⁢L⁢N⁢(z(i,s)(s⁢y⁢n,l-1))∈RDh,

where WQ(l,a)′, WK(l,a)′, and WV(l,a)′ are the matrixes of query, key, and value in the synchronous transformer module, respectively.

Synchronous e self-attention (SSA) scores for $z_{\left(i,s\right)}^{\left(s\,y\,n,l-1\right)}$ in the *a*-th multi-head self-attention unit are given as follows:


(11) α(i,s)(l,a)s⁢y⁢n=σ⁢(q(i,s)(l,a)Dh⋅[k(0,0)(l,a)⁢{k(i,s)(l,a)}s=1,…,S])∈RS,

where $k_{\left(i,s\right)}^{\left(l,a\right)}$ and $q_{\left(i,s\right)}^{\left(l,a\right)}$ denote the corresponding key and query vectors, respectively. The equation shows that the SSA scores are merely computed over the feature map extracted by the same depth-wise convolution. The SSA can calculate the contribution of convolution features changing over time to the final model decision at a specific EEG channel. An intermediate vector $s_{\left(i,s\right)}^{\left(l,a\right)}$ for encoding $z_{\left(i,s\right)}^{\left(s\,y\,n,l-1\right)}$ is given as follows:


(12) $$s_{\left(i,s\right)}^{\left(l,a\right)}=\alpha_{\left(i,0\right)}^{\left(l,a\right)}\,v_{\left(i,0\right)}^{\left(l,a\right)}+\sum_{j=1}^{C}\alpha_{\left(i,j\right)}^{\left(l,a\right)}\,v_{\left(i,j\right)}^{\left(l,a\right)}\in R^{D_{h}}.$$

The encoded feature $z_{\left(i\right)}^{\left(s\,y\,n,l\right)}\in R^{S\times D}$ by the *l*-th block is computed by first concatenating the intermediate vectors from all heads, and the vector concatenation is projected by matrix *W*_*O*_ ∈ *R*^D×*L*^. z(i)(syn,l)′ is the residual connection result of the projection of the intermediate vectors and the $z_{\left(i\right)}^{\left(s\,y\,n,l-1\right)}$ encoded by the preceding block. Finally, the z(i)(syn,l)′ normalized by LN() is passed through a multilayer perceptron (MLP) using the residual connection. The output of the synchronous transformer is represented by *z*_5_ ∈ *R*^C×*S*×D^.

#### 2.3.3. Temporal transformer module

The input of the temporal transformer module is *z*_5_ ∈ *R*^C×*S*×D^. To avoid huge computational complexity, we compress the original temporal dimensionality *D* of *z*_5_ into dimensionality *M*. That is, the 3D matrix z_5_ is first segmented and then averaged into *M* 2D submatrices along the temporal dimension. Each submatrix is represented by ${\text{X}}_{i}^{t\,e\,m\,p}\in R^{S\times C}$ (*i* = 1,2,3,…,*M*) and the *M* submatrices are concatenated to form *X^temp^* ∈ *R*^M×*S*×*C*^. Each submatrix ${\text{X}}_{i}^{t\,e\,m\,p}$ is flattened into a vector Xit′⁢e⁢m⁢p∈RL⁢1, where *L1* is equal to *S*×*C*. The Xit′⁢e⁢m⁢p is defined as a patch and is linearly mapped into a latent vector $z_{\left(i\right)}^{\left(t\,e\,m\,p,0\right)}\in R^{D}$ using a learnable matrix M ∈ *R*^D×*L*^:


(13) z(i)(t⁢e⁢m⁢p,0)=M⁢X(i)t′⁢e⁢m⁢p+e(i)p⁢o⁢s,

where $e_{\left(i\right)}^{p\,o\,s}\in R^{D}$ denotes a positional embedding added to encode the temporal position for each EEG channel changing over the features extracted by different depth-wise convolutional kernels. The module consists of *K* ≥ 1 encoding blocks, each block contains two layers: a multi-head self-attention layer and a position-wise fully connected feed-forward network. The resulting $z_{\left(i\right)}^{\left(t\,e\,m\,p,0\right)}$ is defined as a token representing the inputs of each block, and the $z_{\left(0\right)}^{\left(t\,e\,m\,p,0\right)}$ indicates the classification token. The *l*-th block produces an encoded representation $z_{\left(i\right)}^{\left(t\,e\,m\,p,l\right)}$ for each token in the input sequence by incorporating the attention scores. Specifically, at each block *l*, three core vectors, including $q_{\left(i\right)}^{\left(l,a\right)}$, $k_{\left(i\right)}^{\left(l,a\right)}$, and $v_{\left(i\right)}^{\left(l,a\right)}$ are computed from the representation $z_{\left(i\right)}^{\left(t\,e\,m\,p,l-1\right)}$ encoded by the preceding layer:


(14) q(i)(l,a)=WQ(l,a)″⁢L⁢N⁢(z(i)(t⁢e⁢m⁢p,l-1))∈RDh,

(15) k(i)(l,a)=WK(l,a)″⁢L⁢N⁢(z(i)(t⁢e⁢m⁢p,l-1))∈RDh,

(16) v(i)(l,a)=WV(l,a)″⁢L⁢N⁢(z(i)(t⁢e⁢m⁢p,l-1))∈RDh,

where WQ(l,a)″, WK(l,a)″, and WV(l,a)″ are the matrixes of query, key, and value in the temporal transformer, respectively. The temporal self-attention (TSA) score for $z_{\left(i,s\right)}^{\left(T,l-1\right)}$ in the *a*-th multi-head self-attention unit is given as follows:


(17) α(i)(l,a)t⁢e⁢m⁢p=σ⁢(q(i)(l,a)Dh⋅[k(0)(l,a)⁢{k(i)(l,a)}i=1,…,M])∈RM.

The equation shows that the TSA scores are merely computed over the temporal dimension. The TSA can calculate the contribution of multiple electrode features changing over different convolutional features to the final model decision at a specific time. An intermediate vector $s_{\left(i\right)}^{\left(l,a\right)}$ for encoding *z*^(*temp*,*l*−1)^ is given as follows:


(18) $$s_{\left(i\right)}^{\left(l,a\right)}=\alpha_{\left(i,0\right)}^{\left(l,a\right)}\,v_{\left(i,0\right)}^{\left(l,a\right)}+\sum_{j=1}^{M}\alpha_{\left(i,j\right)}^{\left(l,a\right)}\,v_{\left(i,j\right)}^{\left(l,a\right)}\in R^{D_{h}}.$$

The encoded feature *z*^(*temp*,*l*)^ ∈ *R*^M×*L*1^ by the *l*-th block is computed by first concatenating the intermediate vectors from all heads, and the vector concatenation is projected by matrix *W*_*O*_ ∈ *R*^L1×*L*^. z(temp,l)′ is the residual connection result of the projection of the intermediate vectors and the *z*^(*temp*,*l*−1)^ encoded by the preceding block. Finally, the z(temp,l)′ normalized by LN() is passed through a multilayer perceptron (MLP) using the residual connection. The output of the temporal transformer is represented by O ∈ *R*^M×*L*1^.

### 2.4. EEGformer decoder

The EEGformer is used to extract the temporal, regional, and synchronous characteristics in a unified manner, as well as to deal with various EEG-based brain activity analysis tasks. Unlike the original transformer decoder, which uses a multi-head self-attention mechanism to decode the feature output of the corresponding encoder, we designed a convolution neural network (CNN) to perform the corresponding task. The CNN contains three convolutional layers and one fully connected layer. Specifically, the output O ∈ *R*^M×*L*1^ of the EEGformer encoder is reshaped to X ∈ *R*^S×*C*×*M*^, where *M* is the dimensional length of the encoded temporal feature. The first layer of our EEGformer decoder (with the weights *w*_1_ ∈ *R*^C×1^) linearly combined different convolutional features for normalization across the convolutional dimension. Thus, the output data shape of the first layer is *X*_1_ ∈ *R*^S×*M*^. The motivation to convolve *C* feature maps along the convolutional dimension of X into one is to allow the network to make data–driven decisions about the contribution of different convolutional features to the final model decision. The second layer of our CNN was responsible for combining information across spatial dimensions of X and extracting the entire information while discarding redundancy or noninformative variations. To this end, our CNN convolved X along the spatial dimension using the weights *w*_2_ ∈ *R*^S×*N*^ and returns the plane *X*_2_ ∈ *R*^M×*N*^, where *N* denotes the number of convolutional filters used in the second layer. The third layer halved the dimension and reduced the parameter complexity using the weights *w*_3_ ∈ *R*^(*M*/2)×*N*^ to produce the output plane *X*_3_ ∈ *R*^(*M*/2)×*N*^. The fourth layer of our CNN is a fully connected layer that produced classification results for the brain activity analysis task. The corresponding equation of the loss function is given as follows:


(19) $$L\,o\,s\,s=\frac{1}{D_{n}}\,\sum_{i=1}^{D_{n}}-\log\left(p_{i}\,\left(y_{i}\right)\right)+\lambda \,\left|w\right|$$

where *D*_n_ is the number of data samples in the training dataset, *p*_i_ and *y*_i_ are the prediction results produced by the model and the corresponding ground truth label for the *i*-th data sample, respectively, and λ is the constant of the L1 regularization.

## 3. Experiment results

### 3.1. Experimental setup

For generating the input of the EEGformer and other comparison models, we first extract the raw EEG data of each trial of the three datasets to form data samples and assign the corresponding label to each data sample. Further, we apply a sliding window with the step of *ratio* × *SR* (i.e., SR) on each data sample and generate the final input samples in a non-overlapping manner. The data shape of each input sample is *ratio* × *SR* × *N*_*c*_, and the *N*_*c*_ denotes the number of EEG channels (i.e., 64). The equation for representing the relationship between segment length *T* and the total number of input samples *N* is given as follows:


(20) $$\text{N}=\frac{N_{s\,u\,b}\times E\,P\,P\times T\,P\,E\times T\,L\,P\,T}{r\,a\,t\,i\,o},$$

where *N*_*sub*_ denotes how many subjects joined in the corresponding data collection experiment. Taking the data splitting method for BETA dataset as an example, we remove the EEG data collected during the gaze shifting of 0.5 s guided by a visual cue and an offset of 0.5 s followed by the visual stimulation. The final BETA dataset consists of 11,200 trials and 40 categories. For the first 15 participants and the remaining 55 participants in the BETA dataset, the time length of the flickering visual stimulation in each trial is 2 and 3 s, respectively. When the number of data points of each input sample is 100, meaning the *ratio* is set to 0.4 and the *SR* is equal to 250 Hz, and the time length of each input sample is 0.4 s, the total number of input samples of the BETA dataset for training and testing models is 78,000. Under the same setting, the total number of input samples of the SEED and DepEEG dataset for training and testing models is 514,687 and 42,000.

The state-of-the-art DL models, which have performed well in previous studies, were tested on the three datasets to compare their model performance with ours. In our comparison, we followed the same test procedures for all these methods. The EEGformer and other comparison baselines were trained with a batch size of 64 and Adam optimizer with a learning rate of 0.001. In each transformer module, the number of encoding blocks is equal to three. The models were trained using an early-stop training strategy. Note that all training hypermeters were optimized using the testing data. Pytorch was used to implement these models, which were trained on an NVIDIA Tesla A100 GPU. As mentioned above, we tested our model on three datasets (BETA, SEED, and DepEEG). Fivefold cross-validation was applied to separate the dataset, and the average classification accuracy (ACC) rate, sensitivity (SEN), and specificity (SPE) and the corresponding standard deviation (SD) of them were used as model performance metrics. For multi-category classification, the accuracy rate, which means how many data samples are corrected and labeled out of all the data samples, is calculated as the sum of true positive and true negative divided by the sum of true positive, false positive, false negative, and true negative. The above metrics are calculated using the following formula:


(21) ACC=(TP+TN)/(TP+FP+FN+TN)

(22) SEN=TP/(TP+FN),

(23) SPE=TN/(TN+FP),

where TP denotes true positives, TN denotes true negatives, FP denotes false positives, and FN denotes false negatives.

### 3.2. Comparison baselines

To show the effective model performance of EEGformer, we compared several commonly used DL methods in other studies of EEG-based data analysis tasks, which were target frequency identification, emotion recognition, and depression discrimination. The comparison models are described as follows:

(1) EEGNet (Lawhern et al., 2018). It is a Compact-CNN for EEG-based BCIs. The network starts with a temporal convolution operation to learn frequency filters. The operation is made up of *F*_1_ convolutional filters, and each size equals 1 × *N*, where *N* represents the length of the convolutional filter. It used *D* × *F*_1_ depth-wise convolutional filters to learn frequency-specific spatial filters and the size of each filter is *C* × 1. The separable convolution followed by point-wise convolution was used to learn the summary for each feature map and optimally combine them. The network architecture shows that EEGNet considers temporal and spatial information of EEG signals.
(2) Conv-CCA (Waytowich et al., 2018). It is designed for SSVEPs-based target frequency identification and can be used in other EEG-based classification tasks. Unlike pure DL models, the Conv-CCA uses a signal-CNN with three-layers to transform multiple channel EEGs (*N*_*s*_ × *N*_*c*_ × 1) into a single $\bar{x}$ with a shape of *N*_*s*_ × 1 × 1, where *N*_*s*_ and *N*_*c*_ are the numbers of sampling points and channels, respectively. Another reference CNN with two-layers was used to transform the reference signal (*N*_*s*_ × *N*_*f*_ × *N*_*c*_) into a 2D signal $\bar{Y}$ with a shape of *N*_*s*_ × *N*_*f*_, where *N*_*f*_ is the number of target frequencies. Correlation analysis was used to calculate the coefficients of $\bar{x}$ and each ${\bar{Y}}^{n}$ for all *n* ∈ [1, *N*_*f*_]. A dense layer with *N*_*f*_ units and a softmax activation function was used as the final layer for classification.
(3) 4DCRNN (Shen et al., 2020). It is a DL model known as a four-dimensional (4D) convolutional recurrent neural network that extracts and fuses frequency, spatial and temporal information from raw EEG data to improve model performance of emotion recognition. It is not an end-to-end model for BCI tasks because it requires the Butterworth filter to decompose frequency bands and manually extract differential entropy features from each frequency band. The model input is represented as a 4D structure X ∈ *R*^h×w×d×2T^, where h and w are the height and width of the 2D brain topographical map, respectively, d denotes the number of frequency bands and T denotes the length of the signal segment. CNN was used to extract the frequency and spatial information from each temporal segment of an EEG sample, and long short-term memory (LSTM) was adopted to extract temporal information from CNN outputs.
(4) EmotionNet (Wang et al., 2018). Instead of using 2D convolution filters to extract features from input data, EmotionNet used a 3D convolution filter to learn spatial and temporal features from raw EEG data. The first two layers and the third layer of the model used a 3D convolution filter to learn spatiotemporal and fuse spatial features, respectively. The fourth and fifth layers of the model performed temporal feature extraction using a 2D convolutional filter. The sixth layer of the model is a fully connected layer for dense predictions.
(5) PCRNN (Yang et al., 2018). The model is an end-to-end DL model known as a parallel convolutional recurrent neural network for EEG-based emotion classification tasks. It also takes 3D shape (X ∈ *R*^h×w×T^) of raw EEG data as model input. CNN model was first used to learn spatial feature maps from each 2D map, and the LSTM was used to extract temporal features from the CNN outputs. Note that the CNN and LSTM were organized by a parallel structure to extract the spatial and temporal features from the model input. The outputs of the parallel structure were integrated to classify emotions.

### 3.3. Ablation studies

#### 3.3.1. Effect of the EEGformer decoder constructed by different transformer combination

We conducted an ablation study to show the effectiveness of the EEGformer by constructing the encoder with different combinations of temporal, synchronous, and regional transformers. The classification results (ACC, SPE, SEN, and their corresponding SDs) on the three EEG datasets using different transformer module combinations to construct the EEGformer encoder are shown in Table 2. The table shows that the EEGformer encoder constructed by the combinations of the three transformers achieves the best classification results. For BETA dataset, the average sensitivity, specificity, and accuracy are 69.86, 75.86, and 70.15%, respectively. For SEED dataset, the average sensitivity, specificity, and accuracy are 89.14, 92.75, and 91.58%, respectively. For DepEEG dataset, the average sensitivity, specificity, and accuracy are 77.83, 70.95, and 72.19%, respectively. The result supports our hypothesis that a machine learning method can capture EEG characteristics in a unified manner that can suit the EEG-based brain activity analysis tasks.

**TABLE 2:** Classification results (ACC, SPE, SEN, and their corresponding SDs) on the three EEG datasets by using different transformer module combinations to construct EEGformer encoders.

Combinations	BETA			SEED			DepEEG		
	ACC (%)	SPE (%)	SEN (%)	ACC (%)	SPE (%)	SEN (%)	ACC (%)	SPE (%)	SEN (%)
Reg	41.63 ± 5.91	46.59 ± 3.58	35.67 ± 3.26	76.53 ± 1.68	77.26 ± 2.41	73.58 ± 1.94	58.78 ± 5.21	60.51 ± 2.58	57.25 ± 3.42
Syn	57.29 ± 6.50	62.86 ± 5.89	55.28 ± 4.69	80.12 ± 5.12	82.83 ± 4.65	78.86 ± 2.71	60.12 ± 4.86	65.94 ± 3.59	55.26 ± 4.27
Temp	45.36 ± 7.18	53.38 ± 6.38	43.86 ± 5.68	77.28 ± 4.12	78.29 ± 3.83	76.69 ± 3.82	61.73 ± 4.12	65.82 ± 4.78	60.83 ± 2.65
Temp + Syn	66.52 ± 3.82	70.25 ± 2.97	62.23 ± 4.32	85.36 ± 3.61	88.36 ± 4.75	83.45 ± 2.86	70.15 ± 3.18	68.97 ± 3.56	75.65 ± 4.81
Temp + Reg	59.29 ± 3.27	65.93 ± 2.65	58.79 ± 3.54	80.12 ± 3.19	82.33 ± 2.08	79.16 ± 3.19	65.21 ± 2.89	62.14 ± 4.72	72.31 ± 3.75
Syn + Reg	65.72 ± 2.91	70.85 ± 2.58	61.23 ± 5.12	86.73 ± 2.95	88.04 ± 2.36	83.77 ± 3.76	71.46 ± 2.85	61.96 ± 2.36	75.64 ± 3.19
Temp + Syn + Reg	70.15 ± 2.18	75.86 ± 2.04	69.86 ± 3.29	91.58 ± 2.77	92.75 ± 3.72	89.14 ± 2.98	72.19 ± 2.67	70.95 ± 2.38	77.83 ± 2.15

The table also demonstrates that the EEGformer that contains a synchronous transformer achieves better model performance than the EEGformer without a synchronous transformer. For instance, the EEGformer constructed using a single synchronous transformer outperforms the EEGformer constructed using the other two types of single transformers, with better accuracy of 57.29, 80.12, and 60.12% on BETA, SEED, and DepEEG, respectively. The EEGformer constructed using a transformer pair consisting of a synchronous transformer outperforms the EEGformer constructed using the transformer pair without a synchronous transformer, with better accuracy on the BETA, SEED, and DepEEG datasets. The results indicate the significance of learning spatial distribution characteristics of EEG activity generated by multiple brain regions for the task of SSVEPs-based frequency discrimination. In addition, the EEGformer constructed using synchronous transformer and regional transformer outperforms the EEGformer constructed using other transformer pairs, with better classification results on SEED and DepEEG dataset. On the one hand, the result demonstrates that the convolutional features could represent regional and spatial characteristics of EEH signal well. On the other hand, the result indicates that the integration of the synchronous and regional EEG characteristics improves discrimination ability of our model.

#### 3.3.2. Effect of using 1DCNN or not to construct the EEGformer pipeline

**FIGURE 3:**
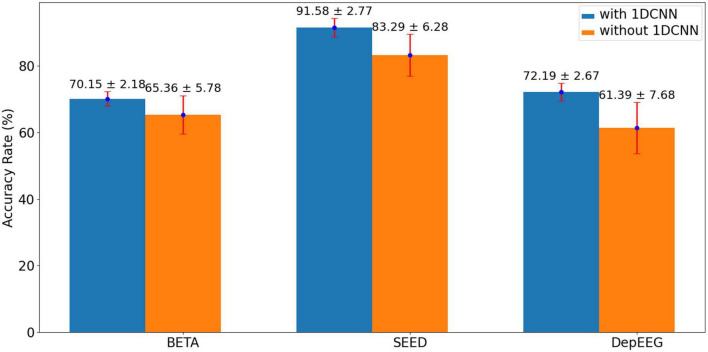
Comparison results of using 1DCNN or not to construct the EEGformer pipeline.

The model performance affected by using 1DCNN or not is validated to show the rationality of using a 1D depth-wise convolutional filter to learn regional characteristics in a completely data–driven manner. Figure 3 compares the results of using 1DCNN or not constructing the EEGformer pipeline. The figure shows that using a 1D depth-wise convolutional filter to learn regional characteristics is beneficial for improving model performance in EEG-based classification tasks.

#### 3.3.3. Effect of EEG channel number on the model performance

Table 3 reports the classification results (ACC, SPE, SEN, and their corresponding SDs) of our model with varying number of EEG channel. The EEG CHN and the corresponding name of brain regions are illustrated as follows: 3 (O1, Oz, and O2), 6 (O1, Oz, O2, POz, PO3, and PO4), 9 (O1, Oz, O2, Pz, PO3, PO5, PO4, PO6, and POz), 32 channels (all channels from occipital, parietal, central-parietal regions and C3, C1, Cz, C2, C4, and FCz) as well as all 64 channels. From the table, we can know that as the EEG CHN increases, the classification results of the EEGformer show an upward trend. This result indicates that although the EEG channels that are placed over the occipital and parietal regions provide perhaps the most informative SSVEP signals, other channels are informative as well. The result also illustrates the data mining ability of our model, which can learn representational features from complex data structure.

**TABLE 3:** Classification results (ACC, SPE, SEN, and their corresponding SDs) of our model is reported versus varying number of channels and 1.0 s of stimulation.

Channel number	BETA			SEED			DepEEG		
	ACC (%)	SPE (%)	SEN (%)	ACC (%)	SPE (%)	SEN (%)	ACC (%)	SPE (%)	SEN (%)
3	42.73 ± 3.60	50.73 ± 5.17	36.83 ± 4.39	69.54 ± 3.86	70.49 ± 2.96	66.76 ± 4.85	51.29 ± 2.99	50.86 ± 3.75	55.71 ± 4.51
6	50.86 ± 4.49	63.69 ± 2.38	55.17 ± 6.73	73.21 ± 2.83	74.62 ± 3.79	73.61 ± 2.73	56.74 ± 3.85	54.14 ± 2.64	60.26 ± 3.29
9	56.52 ± 2.17	70.46 ± 3.96	65.89 ± 5.26	76.37 ± 3.72	77.24 ± 4.21	78.18 ± 3.82	61.21 ± 4.74	59.75 ± 3.82	65.78 ± 2.79
32	65.21 ± 3.05	72.17 ± 2.57	65.36 ± 4.74	85.98 ± 3.16	86.91 ± 2.64	86.27 ± 4.54	68.56 ± 2.38	65.37 ± 3.57	70.39 ± 4.26
64	70.15 ± 2.18	75.86 ± 2.04	69.86 ± 3.29	91.58 ± 2.77	92.75 ± 3.72	89.14 ± 2.98	72.19 ± 2.67	70.95 ± 2.38	77.83 ± 2.15

### 3.4. Comparison studies

**FIGURE 4:**
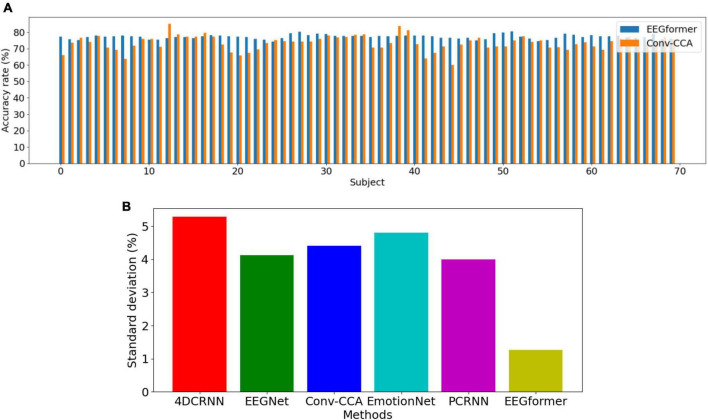
Performance comparison between EEGformer and other five comparison methods using leave-one-subject-out cross-validation method based on BETA dataset. **(A)** Accuracy comparison between EEGformer and Conv-CCA across subjects using BETA dataset. **(B)** Standard deviation comparison between EEGformer and other five comparison methods across subjects using BETA dataset.

**FIGURE 5:**
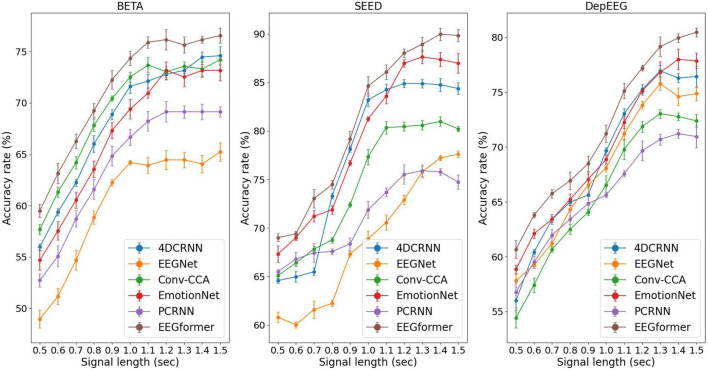
Performance (average ACC ± SD %) of segment length *T* using the EEGformer and other comparable models on the three EEG datasets.

Leave-one-subject-out (LOSO) cross-validation method is utilized to compare the model performance between EEGformer and other five comparison methods. As shown in Figure 4, the upper figure shows accuracy comparison results between EEGformer and Conv-CCA across using BETA dataset, and the lower figure shows standard deviation comparison between EEGformer and other five comparison methods across subjects using BETA dataset. The reason of only choosing Conv-CCA to compare with EEGformer is both of them achieve high accuracy on the BETA dataset. From the Figure 5, we can find that EEGformer achieves the lowest standard deviation among other comparison methods, indicating the proposed method generalizes well on unseen data and potentially requires little to model training and calibration for new users, suitable for SSVEP classification tasks.

1. Accuracy comparison between EEGformer and Conv-CCA across subjects using BETA dataset.
2. Standard deviation comparison between EEGformer and other five comparison methods across subjects using BETA dataset.

Furthermore, according to the SSVEP studies, they pursue a higher information transfer rate by not using long EEG segments to execute the target frequency identification task. The model performance can be improved by increasing the segment length *T* because longer EEG segments contain more information about brain activity. Therefore, we investigated the impact of segment length *T* ranges [0.5, 0.6, 0.7, 0.8, 0.9, 1.0, 1.1, 1.2, 1.3, 1.4, 1.5] on model performance. The performance (average ACC and SD) of segment length *T* using the EEGformer and other comparable models on the three EEG datasets are shown in Figure 5. The figure shows that our model achieves the best accuracy rate across the three datasets. For other comparison baseline models, the model performance reduces in some cases if the segment length *T* exceeds 1.2 s. The model performance of the EEGformer on the three datasets showed an increasing trend as the segment length *T* increases, indicating that our method can extract inherent temporal information from EEG and is unaffected by segment length. In addition, the model performance of 4DRCNN and EmotionNet outperforms the performance of other comparison baselines. Because 4DRCNN and the EmotionNet are models that learn spatiotemporal features simultaneously, this operation may facilitate the DL model to learn better feature representation of EEG regional and synchronous characteristics.

## 4. Discussion

The abovementioned ablation and comparison studies show the rationality of our EEGformer architecture and demonstrate that our model performs outperforms other comparison baselines. This section covers several noteworthy points and future works:

(1) The unified manner, sequentially maps an input sequence into an abstract continuous representation that holds temporal, convolutional, and spatial information of that input outperforms the 2D and 3D structures that integrate frequency, spatial and temporal information of EEG. The EEGformer achieved the highest accuracy rate compared with other comparison baselines, which could be due to the unified EEG characteristics learning manner. Compared with 4DRCNN, which requires the user to manually extract frequency information from raw EEG data and use it as model input, our model is an end-to-end deep method because it uses depth-wise 1DCNN to learn the feature in an EEG-channel-wise manner. In the EEGformer encoder, we sequentially encode the convolutional results generated by the 1DCNN from temporal, convolutional, and spatial dimensions. The temporal, regional, and synchronous transformers were responsible for learning the temporal, regional, and synchronous characteristics of EEG signals. This type of feature learning strategy contains more cues of EEG characteristics than other model structures and performs better than them.
(2) EEG signals are well-known to exhibit data statistics that can drastically change from one subject to another in various aspects (e.g., regional characteristics), but also share similarities in certain other aspects (e.g., synchronous characteristics). To exploit the commonalities while tackling variations, we require a large data sample to train the model and improve its generalization ability. However, the performance of a DL model is always affected by the dataset size. Compare with the dataset size in the computer vision studies, researchers find it difficult to collect a dataset with a similar size in EEG-based clinical studies. Therefore, increasing the number of EEG datasets used for training DL models is crucial to reduce the influence of small dataset size on model performance. To this end, many studies separate the EEG signal collected in a trial into several segments and label them with the same label. Those segments were then used in cross-subject and within-subject classifications, which are two commonly used experimental designs, to execute model training and validate model performance. Meanwhile, those studies also designed model training strategies to improve the model generalization ability. For instance, Guney et al. (2021) trained their model in two stages: the first stage trains globally with all the available data from all the subjects, and then the second stage fine-tunes the model individually using the data of each subject separately. In the future, we can also design a training strategy to reduce the influence of small dataset size on model performance.
(3) Although the experimental results demonstrated that learning temporal, regional, and spatial characteristics in a unified manner facilitates the EEGformer to achieve promising classification performance across three EEG datasets, this result might be unable to provide strong support for clinical treatment that is associated with EEG biomarkers. Because DL methods are essentially considered black boxes, we require novel methods to open the box and visualize the feature learned by the DL model. To this end, an emerging technique known as explainable artificial intelligence (AI) enables the understanding of how DL methods work and what drives their decision-making. The competitive model performance of DL methods and the explainable AI provided a promising way to support effective EEG-based brain activity analysis. By using the explainable AI method, we could visualize the form of the temporal, regional, and spatial characteristics learned by the EEGformer and use it to connect with BFC, as well as perform brain activity analysis.

## 5. Conclusion

In this study, we proposed a transformer–based EEG analysis model known as EEGformer to capture EEG characteristics in a unified manner. The EEGformer consists of 1DCNN, an EEGformer encoder (sequentially constructed by three components: regional, synchronous, and temporal transformers), and an EEGformer decoder. We conducted ablation studies to demonstrate the rationality of the EEG former. The results not only supported our hypothesis that a machine learning method capable of capturing the EEG characteristics in a unified manner can be applied to EEG-based brain activity analysis tasks but also demonstrated that convolutional features could accurately represent regional and spatial characteristics of EEG signals. The LOSO cross-validation method is utilized to compare the model performance between EEGformer and other five comparison methods, the result shows the proposed method generalizes well on unseen data and potentially requires little to model training and calibration for new users, suitable for SSVEP classification tasks. We also investigate the impact of segment length *T* on model performance, and the results show that our method can extract inherent temporal information from EEG and is unaffected by the segment length. The proposed EEGformer outperforms the comparison models, which perform well in other studies on the three EEG datasets.

## Data availability statement

Publicly available datasets were analyzed in this study. This data can be found here: http://bci.med.tsinghua.edu.cn/download.html and https://bcmi.sjtu.edu.cn/home/seed/seed.html.

## Ethics statement

The studies involving human participants were reviewed and approved by the Institutional Review Board of Beijing Anding Hospital of Capital Medical University. The patients/participants provided their written informed consent to participate in the data collection. Written informed consent was obtained from the individual(s) for the publication of any potentially identifiable data included in this article.

## Author contributions

ZW, ML, and WD contributed to the conception and design of the study. SL and ML performed the data analysis. ZW and WD drafted the manuscript. JH and HT participated in editing the manuscript. All authors contributed to the article and approved the submitted version.
